# Impact of three multimodal countrywide campaigns to promote hand hygiene in Belgian hospitals

**DOI:** 10.1186/1753-6561-5-S6-O63

**Published:** 2011-06-29

**Authors:** AC Simon, M Costers

**Affiliations:** 1 Microbiology/Infection Control, Cliniques Universitaires Saint-Luc, Belgium; 2 Belgian Antibiotic Policy Coordination Committee, Federal Public Service for Public Health, Food Chain Safety and Environment, Brussels, Belgium

## Introduction / objectives

In Belgium three multimodal, country-wide hand hygiene campaigns were organised between 2005 and 2009 with financial support of the federal government. The objective of these campaigns was to raise awareness among healthcare workers (HCWs) in hospitals and to increase their adherence to good hand hygiene practices.

## Methods

In order to increase adherence audit with performance feedback of their compliance and use of alcohol-based hand rubs (ABHR), education, reminders on the workplace and patient empowerment were used.

## Results

Voluntary participation and commitment of hospitals was excellent, with participation rates of 95% for acute care hospitals, 65% for chronic care hospitals and 60% for psychiatric hospitals, for all campaigns.

Each of the three national hand hygiene campaigns resulted in a significant increase in hand hygiene compliance among HCWs and a higher consumption of ABHR. Hand hygiene compliance, measured by direct observation, increased significantly from 49% to 69% during the first campaign, from 53% to 69% during the second campaign and from 58% to 69% during the third campaign. In view of these positive outcomes, hand hygiene campaigns will be repeated have now become a priority for our government. every two years.

## Conclusion

Government support, one of the WHO’s key recommendations was one of the most important reasons for success of the Belgian national campaigns.

The next campaign is ongoing (winter 2010-2011). The main focus of this fourth campaign is the physician’s behaviour. The results of hand hygiene compliance among physicians show us that there is more place for improvement.

## Disclosure of interest

None declared.

